# O.6.2-1 Contextual influences on sport promotion policy in The Netherlands: why and how the COVID-19 pandemic led to changes in local sport promotion policies

**DOI:** 10.1093/eurpub/ckad133.271

**Published:** 2023-09-11

**Authors:** Linda Ooms, Remco Hoekman

**Affiliations:** Mulier Institute, The Netherlands; Mulier Institute, The Netherlands; Department of Sociology, Radboud University, The Netherlands; l.ooms@mulierinstituut.nl

## Abstract

**Purpose:**

The Dutch government implemented various public health mandates to reduce the spread of the Covid-19 virus, such as self-quarantining, social distancing, closure of schools and sport facilities. This also impacted the sport sector and local sport promotion policies. The aim of the research was to get insight into why and how the Covid-19 pandemic and related restrictions influenced local sport promotion policy.

**Methods:**

At the end of the Covid-19 pandemic, online questionnaires were sent to local sport managers (LSMs, n = 352, response 26%). These persons are responsible for the development of sport promotion policy within a municipality. In addition, online group interviews (n = 11) were conducted with LSMs and neighborhood sport coaches (NSCs). The latter are important implementers of sport promotion policy activities. A socio-ecological model of local sport promotion in the Netherlands was used as a theoretical framework.

**Results:**

LSMs and NSCs reported that the Covid-19 pandemic and restrictions led to (some) changes in local sport promotion policy related to the facilities and public space (hardware), activity providers (orgware) and organized activities (software). In most cases, this did not lead to totally new policy, but there was a shift in focus related to the importance or urgency. For example, the growing importance of facilitating sport in public space and using sport as a means to promote health. Moreover, the Covid-19 pandemic and additional financial resources (Covid-19 recovery funds) from the national government enhanced the implementation of sport promotion activities, especially for children, adolescents and older adults.

**Conclusions:**

The Covid-19 pandemic and restrictions influenced sport promotion policy (activities) in a positive way at the national level, the local level and within the municipal office. The general awareness of the importance of sport for health helped in legitimizing sport promotion policy and activities at all levels. However, it is questionable whether these changes are structural, especially when additional funding options cease. Considering the increased inequalities in sport and physical activity participation, it is important there remains attention for sport promotion of specific inactive target groups.

**Funding source:**

This study was funded by ZonMw, The Netherlands, grant number [10430032010016].

